# Molecular epidemiology of resistance to antimalarial drugs in the Greater Mekong subregion: an observational study

**DOI:** 10.1016/S1473-3099(20)30228-0

**Published:** 2020-12

**Authors:** Mallika Imwong, Mehul Dhorda, Kyaw Myo Tun, Aung Myint Thu, Aung Pyae Phyo, Stephane Proux, Kanokon Suwannasin, Chanon Kunasol, Suttipat Srisutham, Jureeporn Duanguppama, Ranitha Vongpromek, Cholrawee Promnarate, Aungkana Saejeng, Nardlada Khantikul, Rungniran Sugaram, Supinya Thanapongpichat, Nongyao Sawangjaroen, Kreepol Sutawong, Kay Thwe Han, Ye Htut, Khin Linn, Aye Aye Win, Tin M Hlaing, Rob W van der Pluijm, Mayfong Mayxay, Tiengkham Pongvongsa, Koukeo Phommasone, Rupam Tripura, Thomas J Peto, Lorenz von Seidlein, Chea Nguon, Dysoley Lek, Xin Hui S Chan, Huy Rekol, Rithea Leang, Cheah Huch, Dominic P Kwiatkowski, Olivo Miotto, Elizabeth A Ashley, Myat Phone Kyaw, Sasithon Pukrittayakamee, Nicholas P J Day, Arjen M Dondorp, Frank M Smithuis, Francois H Nosten, Nicholas J White

**Affiliations:** a Department of Molecular Tropical Medicine and Genetics, Faculty of Tropical Medicine, Mahidol University, Bangkok, Thailand; b Mahidol–Oxford Tropical Medicine Research Unit, Faculty of Tropical Medicine, Mahidol University, Bangkok, Thailand; c Department of Clinical Tropical Medicine, Faculty of Tropical Medicine, Mahidol University, Bangkok, Thailand; d Worldwide Antimalarial Resistance Network, Bangkok, Thailand; e Department of Preventive and Social Medicine, Defence Services Medical Academy, Yangon, Myanmar; f Shoklo Malaria Research Unit, Mahidol–Oxford Tropical Medicine Research Unit, Faculty of Tropical Medicine, Mahidol University, Mae Sot, Thailand; g Myanmar Oxford Clinical Research Unit, Yangon, Myanmar; h Bureau of Vector-borne Diseases, Department of Disease Control, Ministry of Public Health, Nonthaburi, Thailand; i Office of Disease Prevention and Control Region 1, Chiang Mai, Thailand; j Faculty of Medical Technology, Prince of Songkla University, Songkhla, Thailand; k Department of Microbiology, Faculty of Science, Prince of Songkla University, Songkhla, Thailand; l Buntharik Hospital, Amphoe Buntharik, Ubon Ratchathani, Thailand; m Department of Medical Research, Ministry of Health and Sports, Yangon, Myanmar; n Department of Tropical and Infectious Diseases, University of Medicine 1, Yangon, Myanmar; o Defence Services Medical Research Centre, Naypyitaw, Myanmar; p Centre for Tropical Medicine and Global Health, Nuffield Department of Medicine, University of Oxford, Oxford, UK; q Institute of Research and Education Development, University of Health Sciences, Ministry of Health, Vientiane, Laos; r Lao–Oxford–Mahosot Hospital–Wellcome Trust Research Unit, Vientiane, Laos; s Savannakhet Provincial Health Department, Phonsavangnuea village, Kaysone-Phomvihan district, Savannakhet, Laos; t National Center for Parasitology, Entomology, and Malaria Control, Phnom Penh, Cambodia; u Wellcome Sanger Institute, Hinxton, UK; v Medical Research Council Centre for Genomics and Global Health, Big Data Institute, University of Oxford, Oxford, UK; w Department of Medical Research, Myanmar Health Network Organization, Yangon, Myanmar; x The Royal Society of Thailand, Dusit, Bangkok, Thailand; y Medical Action Myanmar, Yangon, Myanmar

## Abstract

**Background:**

The Greater Mekong subregion is a recurrent source of antimalarial drug resistance in *Plasmodium falciparum* malaria. This study aimed to characterise the extent and spread of resistance across this entire region between 2007 and 2018.

**Methods:**

*P falciparum* isolates from Myanmar, Thailand, Laos, and Cambodia were obtained from clinical trials and epidemiological studies done between Jan 1, 2007, and Dec 31, 2018, and were genotyped for molecular markers (*pfkelch, pfcrt, pfplasmepsin2*, and *pfmdr1*) of antimalarial drug resistance. Genetic relatedness was assessed using microsatellite and single nucleotide polymorphism typing of flanking sequences around target genes.

**Findings:**

10 632 isolates were genotyped. A single long *pfkelch* Cys580Tyr haplotype (from −50 kb to +31·5 kb) conferring artemisinin resistance (PfPailin) now dominates across the eastern Greater Mekong subregion. Piperaquine resistance associated with *pfplasmepsin2* gene amplification and mutations in *pfcrt* downstream of the Lys76Thr chloroquine resistance locus has also developed. On the Thailand–Myanmar border a different *pfkelch* Cys580Tyr lineage rose to high frequencies before it was eliminated. Elsewhere in Myanmar the Cys580Tyr allele remains widespread at low allele frequencies. Meanwhile a single artemisinin-resistant *pfkelch* Phe446Ile haplotype has spread across Myanmar. Despite intense use of dihydroartemisinin–piperaquine in Kayin state, eastern Myanmar, both in treatment and mass drug administrations, no selection of piperaquine resistance markers was observed. *pfmdr1* amplification, a marker of resistance to mefloquine, remains at low prevalence across the entire region.

**Interpretation:**

Artemisinin resistance in *P falciparum* is now prevalent across the Greater Mekong subregion. In the eastern Greater Mekong subregion a multidrug resistant *P falciparum* lineage (PfPailin) dominates. In Myanmar a long *pfkelch* Phe446Ile haplotype has spread widely but, by contrast with the eastern Greater Mekong subregion, there is no indication of artemisinin combination therapy (ACT) partner drug resistance from genotyping known markers, and no evidence of spread of ACT resistant *P falciparum* from the east to the west. There is still a window of opportunity to prevent global spread of ACT resistance.

**Funding:**

Thailand Science Research and Innovation, Initiative 5%, Expertise France, Wellcome Trust.

## Introduction

The emergence and uncontrolled spread of artemisinin-resistant *Plasmodium falciparum* parasites in the Greater Mekong subregion in the past 13 years represents a serious potential threat to malaria control in the Indian subcontinent and Africa. Artemisinin resistance compromises artemisinin combination therapies (ACTs), the first-line treatment for falciparum malaria.^1^ Artemisinin resistance increases the risk of ACT treatment failure and therefore selects for partner drug resistance.^2^, ^3^ Artesunate–mefloquine has failed in east Myanmar and dihydroartemisinin–piperaquine has failed across Cambodia, northeast Thailand, and southern Vietnam.^4^, ^5^, ^6^ Mutations in the gene encoding a Kelch protein (*pfkelch*; PF3D7_1343700) on *P falciparum* chromosome 13 are strongly associated with artemisinin resistance,^7^ resulting in reduced ring stage susceptibility and slow parasite clearance. *pfplasmepsin2-3* (PF3D7_1408000) amplification and mutations in the *P falciparum* chloroquine resistance transporter (*pfcrt*; PF3D7_0709000) downstream of the 4-aminoquinoline resistance locus (positions 72–76 with Lys76Thr) have both been associated with piperaquine resistance.^5^, ^8^, ^9^, ^10^, ^11^ *pfmdr1* (PF3D7_0523000) amplification is a well established marker of mefloquine resistance,^4^, ^12^ which also contributes to reduced lumefantrine susceptibility in southeast Asia.^13^ We aimed to assess these resistance markers in parasite isolates obtained in large studies done across the Greater Mekong subregion and to assess genetic relatedness and thus haplotype spread.

**Research in context**

**Evidence before this study**

We searched PubMed on Dec 18, 2019, using the terms “artemisinin resistance” or “piperaquine resistance”, and “genotype”, and either “Cambodia”, ”Thailand”, “Myanmar” or “Laos” without any date or language restrictions. This search identified 36 non-duplicate articles, of which 26 contained information on the molecular epidemiology of artemisinin-resistant or piperaquine-resistant *Plasmodium falciparum*. Several articles described the finding of multiple different *pfkelch13* mutations conferring artemisinin resistance in *P falciparum* malaria parasites isolated across the Greater Mekong subregion, with reports by Imwong and colleagues and Amato and colleagues describing the emergence of a dominant parasite lineage in the eastern Greater Mekong subregion (comprising eastern Thailand, Cambodia, Laos, and the Southern Vietnam geographical region). *pfplasmepsin2* gene amplification and *pfcrt* mutations linked with piperaquine resistance and high rates of treatment failure were associated with this same lineage. The possibility of resistance spreading from the eastern to the western Greater Mekong subregion (comprising western Thailand and Myanmar), the spread of resistant lineages within Myanmar, and the role of intensive dihydroartemisinin–piperaquine deployment, particularly in mass drug administrations, in the selection of antimalarial drug resistance has not been explored.

**Added value of this study**

This very large molecular epidemiology study done over 12 years describes the evolution and spread of antimalarial drug resistance across the entire Greater Mekong subregion. It shows that multiple soft selective sweeps with different *pfkelch* mutant (artemisinin resistant) parasite lineages have been followed by hard selective sweeps by presumably fitter parasites. These increasingly dominant lineages have emerged in Myanmar and the eastern Greater Mekong subregion, but they differ from one another, and there is no evidence of spread from the eastern to the western Greater Mekong subregion. There is also no evidence for piperaquine resistance in the western Greater Mekong subregion. Intense targeted elimination activities using dihydroartemisinin–piperaquine in the treatment of symptomatic malaria and mass treatments have been successful and did not select for resistance.

**Implications of all the available evidence**

Outside the eastern Greater Mekong subregion there is still a window of opportunity to halt the spread of artemisinin resistance westward to India and Africa by using dihydroartemisinin–piperaquine in targeted malaria elimination activities.

## Methods

### Study design and participants

As part of studies^3^, ^11^, ^15^, ^16^, ^17^, ^18^, ^19^, ^20^, ^21^, ^22^, ^23^, ^24^, ^25^ on the epidemiology, treatment, and targeted elimination of artemisinin-resistant malaria done between Jan 1, 2007, and Dec 31, 2018, across the Greater Mekong subregion, venous blood samples, filter paper blood spots, and completed rapid diagnostic test strips were collected from patients presenting with microscopy or rapid test-confirmed uncomplicated falciparum malaria and from healthy participants in surveys of villages where targeted malaria elimination activities were planned (appendix pp 10–11).

### Procedures

DNA was extracted from either dried blood spots, completed malaria rapid diagnostic test strips, or frozen whole blood samples, by standard methods at the Faculty of Tropical Medicine, Mahidol University, Bangkok, Thailand. DNA was purified with QIAamp DNA Mini kits (QIAGEN; Düsseldorf, Germany).

Polymorphism in the *pfkelch* gene was examined by nested PCR amplification covering the propeller region of the gene^3^ and Sanger sequencing (ABI Sequencer; Macrogen, Seoul, South Korea). The sequences were aligned against the 3D7 reference strain *pfkelch* gene (putative PF13_0238; NCBI sequence XM_001350122·1) using Bioedit software. *pfcrt* was amplified from the DNA template using nested PCR covering exons 1 and 2 (amino acids 1–120) and Sanger sequenced. A restriction fragment length polymorphism assay was developed to assess previously identified *pfcrt* mutations:^10^, ^25^ Phe145Ile, Ile218Phe, Asn326Ser, Met343Ile/Leu, and Gly353Val. Digestion fragments were analysed on a 3% agarose gel. For quality control a random third of all PCR products were sequenced.

*pfplasmepsin2* and *pfmdr1* copy numbers were quantified using a Taqman Relative quantitative real-time PCR on a Corbett Rotor-Gene Q (Corbett Research; Mortlake, NSW, Australia) as described previously.^9^, ^11^ Amplification was done in triplicate as multiplex PCR using Quantitec Multiplex PCR noROX (QIAGEN; Düsseldorf, Germany). Copy number was calculated from the cycle threshold (Ct) with the following formula: 2^−ddCt^, where ddCt denotes the difference between the unknown sample dCt and the reference sample dCt. A copy number cutoff of 1·5 defined *pfplasmepsin2* and *pfmdr1* amplification. Reactions were repeated if either profile did not conform to exponential kinetics, ddCt spread was more than 1·5, or the Ct value was more than 35. Samples with an estimated copy number more than 1·3 were also retested once, the second result counting as final.

Nine microsatellite markers and five single nucleotide polymorphisms (SNPs) in the flanking regions each side of the *pfkelch* gene spaced from −56 kb to +225 kb were assessed.^14^, ^15^ The microsatellite PCR generated product lengths were compared with internal size standards (Genescan 500 LIZ) on an ABI 3100 Genetic Analyzer (Macrogen, Seoul, South Korea). Genescan and Genotyper software was used to measure allele lengths and quantify peak heights. SNPs were examined by PCR amplification^15^ and Sanger sequencing of the gene (ABI Sequencer; Macrogen, Seoul, South Korea). Again, negative controls without template were included in each amplification run. A subset of ten samples was analysed in triplicate to assess intra-assay consistency. Samples with multiple genotypes were excluded from the statistical analysis.

15 microsatellite markers in the flanking regions of *pfplasmepsin2* were designed and developed covering 510 kb (from −243 kb at the 5ʹ end to +267 kb at the 3ʹ end of the gene; appendix pp 2, 12). Markers were amplified by PCR with 35 cycles of 1 min denaturation at 95°C, 1 min annealing at 55°C, and 1 min extension at 72°C.

To identify the size and gene content of the amplified chromosomal regions, we developed 37 real-time PCR assays to measure the copy numbers of genes covering −61 kb at 5ʹ to +130 kb at the 3ʹ end of *pfplasmepsin2* on chromosome 14 (appendix pp 2, 13–14). A nested PCR was developed to identify the precise position of the *pfplasmepsin2* amplicon chromosomal breakage points. Primer pairs amplified across unique junctions of multiple copy *pfplasmepsin2* but produced no product in samples with a single copy (appendix p 15).

### Statistical analysis

Genetic variation at each microsatellite locus or expected heterozygosity (H_e_) was assessed using H_e_=(n/[n–1])(1–Σp_i_^2^), with sampling variance defined as 2(n–1/n^3^[2(n–2)]Σp_i_^3^–Σp_i_^2^)^2^, where n is the sample size and p_i_ the frequency of the allele at position i. Heterozygosity at each location was compared with wild-type sequences using Fisher's least significant difference and Mann-Whitney U tests. Proportions were compared with χ^2^ or Fisher's exact test.

To order the isolates by similarity, we computed pairwise identity-by-state, defined as the proportion of identical alleles over all markers typed (not missing for both isolates). For markers where two or more alleles were observed, phased haplotypes were not estimated but instead identity-by-state was called if at least one allele was shared in both isolates. Hierarchical agglomerative clustering was applied to the distance matrix, where the pairwise distance was defined as one minus the proportion identical-by-state. The output isolate ordering was used to plot the observed marker data using previously published software.^26^

### Role of the funding source

The funders of the study had no role in study design, data collection, data analysis, data interpretation, or writing of the report. The corresponding author had full access to all the data in the study and had final responsibility for the decision to submit for publication.

## Results

14 509 positive samples were obtained (appendix pp 1, 10–11). 10 632 samples had sufficient DNA for genotyping, of which 2420 (23%) were from used rapid diagnostic tests, 4912 (46%) were from filter paper dried blood spots (DBS), 3097 (29%) were from frozen packed red cell samples, and 203 (2%) were from frozen whole blood samples.

**Figure 1:**
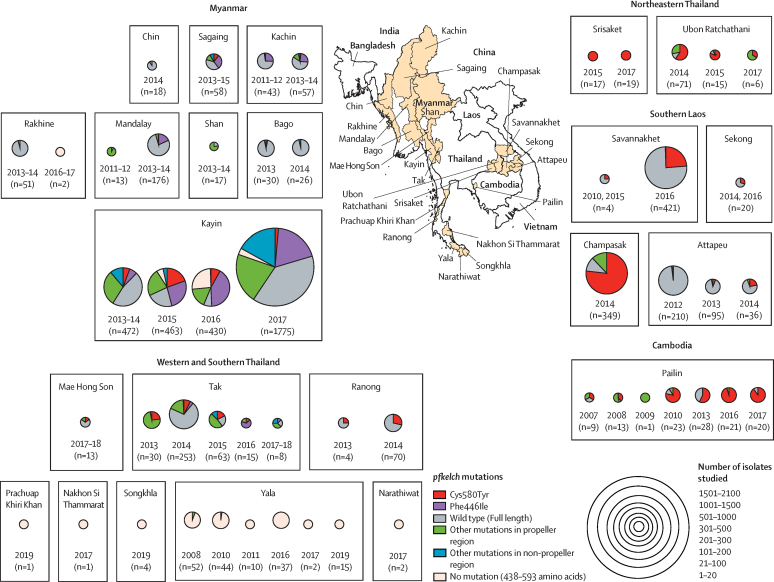
Frequency distributions of mutations found in the *pfkelch* gene in *Plasmodium falciparum* isolates obtained from four countries of the Greater Mekong subregion, 2007–18

**Figure 2:**
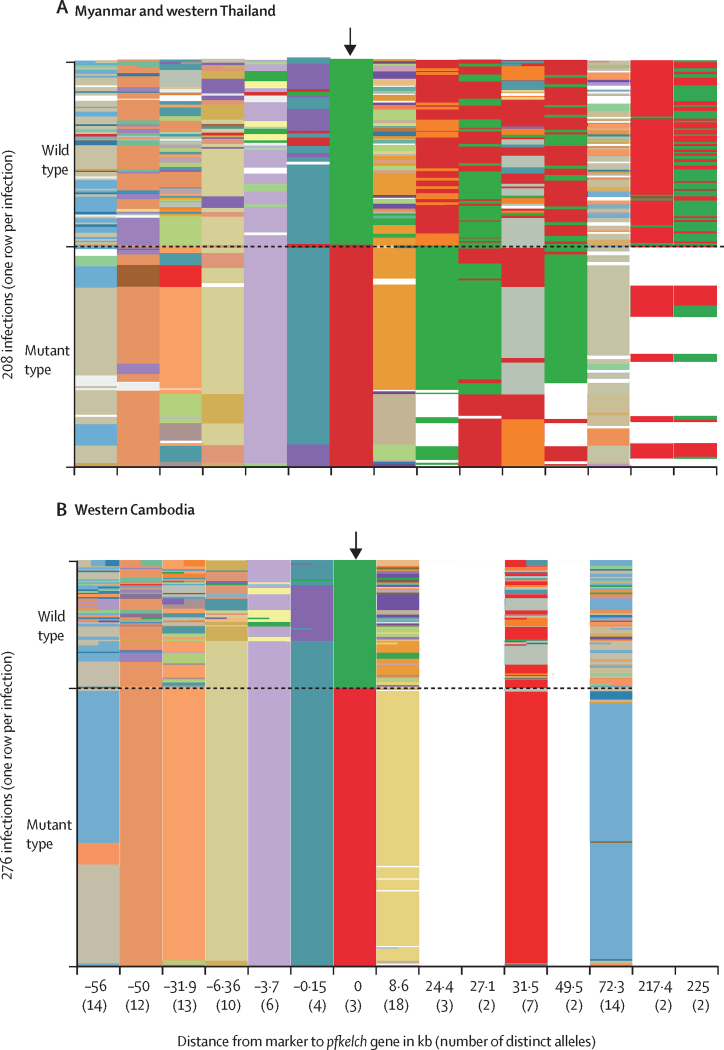
Polyallelic marker data from all Cys580Tyr mutant *Plasmodium falciparum* isolates (A) Data from all mutant isolates from Myanmar (n=113) with matched wild-type isolates (n=95). (B) Data from all mutant isolates from western Cambodia (n=189) with matched wild-type isolates (n=87). Shown are data at position 580 on the *pfkelch* gene (0 on the x-axis) and in an interval from −56 kb to +225 kb surrounding the *pfkelch* gene. The colours correspond to the different alleles, whereby an independent colouring scheme was applied to each polyallelic marker separately. The colour scheme is based on all observed alleles for all samples from the Greater Mekong subregion (not only those shown in this figure), so that comparisons can be made across figures. When multiple alleles were observed at a single locus, the column is broken into subcolumns with the corresponding colours. White corresponds to missing data. For the *pfkelch* gene, green is wild-type and red is Cys580Tyr. The number of distinct alleles observed in all the data for each marker is given by the number in parentheses.

**Figure 3:**
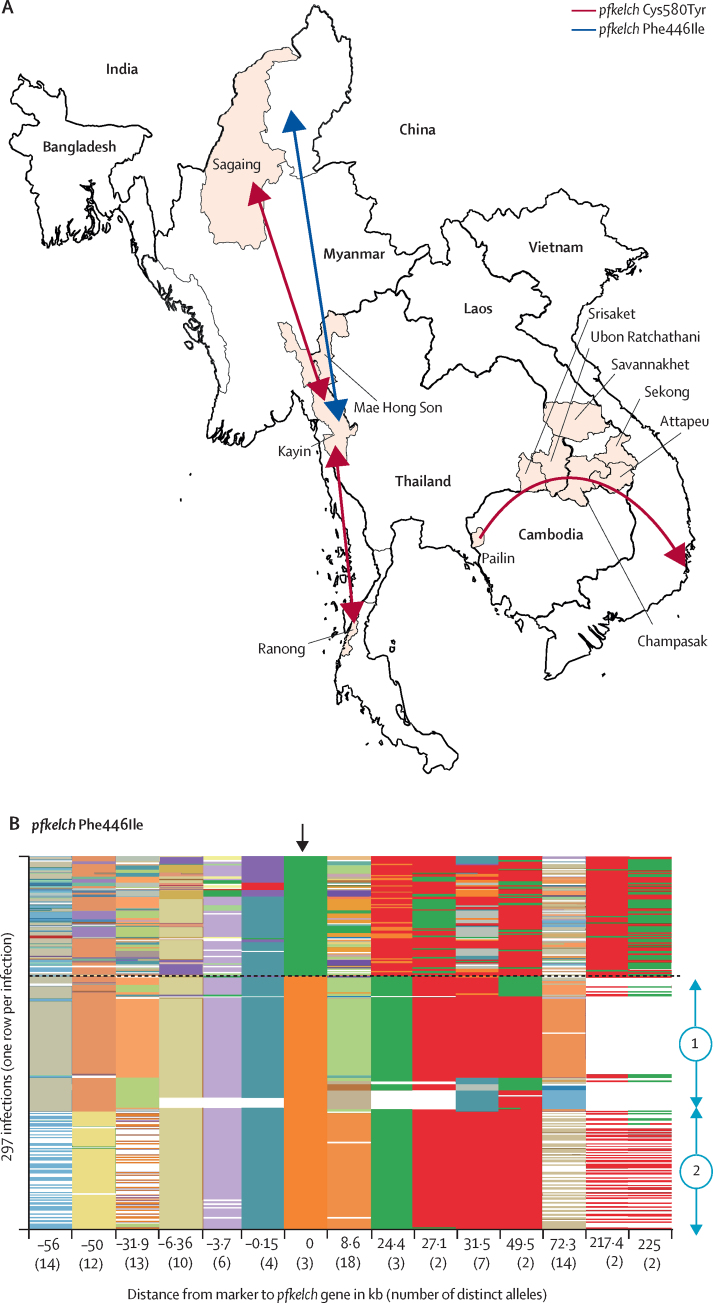
The spread of *pfkelch* haplotypes across the Greater Mekong subregion (A) Map of the Greater Mekong subregion showing the spread of *pfkelch* haplotypes. The single long *pfkelch* Cys580Tyr haplotype (from −50 kb to +31·5 kb; PfPailin; red arrows) emerged in western Cambodia in 2008 and spread across the eastern Greater Mekong subregion.^15^, ^24^ Cys580Tyr bearing parasites of a different lineage have spread widely in Myanmar but have not dominated, except on the Thailand–Myanmar border. There the previously dominant lineage^27^ almost disappeared as falciparum malaria was eliminated by intensive targeted activities in Kayin state. A single *pfkelch* Phe446Ile haplotype (blue arrows), which probably originated in the north, has spread widely across Myanmar. Two haplotypes were evident, although one has predominated since 2017. (B) Polyallelic marker data (single nucleotide polymorphisms and microsatellites) at the amino acid 446 position on the *pfkelch* gene (0 on the x-axis), and in an interval of −56 kb to +225 kb surrounding the *pfkelch* gene. This plot shows the marker data for all isolates (one row per isolate) with the Phe446Ile mutation (n=202) found in Myanmar and 95 matched wild-type isolates. For *pfkelch*, green is wild-type and orange is Phe446Ile.

5633 *P falciparum* isolates collected between Jan 1, 2007, and Dec 31, 2018, were sequenced for *pfkelch* mutations. Two genotypes, the Cys580Tyr mutation in the eastern Greater Mekong subregion and, to a lesser extent, the Phe446Ile mutation in Myanmar, have come to predominate with a corresponding decline in the proportions of other propeller mutations (Figure 1, Figure 2, Figure 3). In addition to the eastern Greater Mekong subregion, the Cys580Tyr allele was found also in Ranong, southwest Thailand adjacent to Myanmar (21 [28%] of 74), and in Mae Hong Son (two [15%] of 13), and Mae Sot (39 [11%] of 369), in northwestern Thailand along the Myanmar border (figure 1; appendix pp 3–4). In western Cambodia the proportion of Cys580Tyr isolates has increased:^15^ Cys580Tyr was present in three (33%) of nine of isolates in 2007 compared with 18 (90%) of 20 in 2017 (p=0·0043; figure 1). Within Myanmar, the Cys580Tyr mutation was distributed widely at low frequencies in Kayin (177 [5%] of 3140), Kachin (one [1%] of 100), and Sagaing (six [10%] of 58) states. The Phe446Ile allele was observed only in the western Greater Mekong subregion (figure 1). The prevalence of Phe446Ile among the *P falciparum* isolates was highest towards the north of Myanmar.^16^ Phe446Ile was also common inside Kayin state but prevalence was lower at the Thai border, and it was not found in 2014 in Ranong, Thailand, near the southern tip of Myanmar.

Comparison of the Cys580Tyr flanking sequences (n=113) with contemporary *pfkelch* wild-type isolates (n=95) in the western Greater Mekong subregion between 2007 and 2017 revealed a marked reduction in the heterozygosity of mutant infections over time (H_e_ 0·220, standard error [SE] 0·002) suggesting a selective sweep (figure 2). In the subset of Cys580Tyr isolates from 2016 and 2017 (n=74) the sweep was more evident, with a mean H_e_ of 0·130 (SE 0·001) versus 0·696 (0·001) in contemporary wild-type infections (p<0·0001). Flanking sequence variation was diminished for about 50 kb either side of the Cys580Tyr *pfkelch* gene (appendix pp 3–4). Comparison of the flanking haplotypes showed that the Cys580Tyr allele in the northwest (Mae Hong Son, n=2) and southwest of Thailand (Ranong, 1000 km to the south, n=13) shared a common origin with the Cys580Tyr alleles observed in northwestern Myanmar (Sagaing, 1800 km north of Ranong, n=6) and eastern Myanmar (Kayin, n=47). This haplotype has not dominated except on the Thai border, where a focus has been eliminated by targeted elimination activities. This common haplotype was genetically remote from the previously characterised PfPailin Cys580Tyr haplotype,^15^ which has spread across the eastern Greater Mekong subregion (Figure 2, Figure 3). Reduced flanking sequence variation (H_e_ <0·1) was observed in a smaller segment of the genome surrounding Cys580Tyr in Myanmar in 2016–17 than in earlier years (2010–15), suggesting continued recombination after the initial selection event.

Flanking sequences around the *pfkelch* Phe446Ile allele were assessed in 202 samples from Myanmar obtained between Jan 1, 2014, and Dec 31, 2017. In 2014–16, at least two *pfkelch* Phe446Ile haplotypes were observed in Kayin, Kachin, and Sagaing states (n=90), but by 2017, one predominated and had spread across more than 1000 km from the northwest to the east of Myanmar (Sagaing n=13; Kayin n=99) with a mean H_e_ of 0·207 (SE 0·066; appendix p 5). The size of the region of the genome around the Phe446Ile and Cys580Tyr alleles, in which diversity was reduced, was roughly similar (ie, about 50 kb either side of the gene). By comparison, diversity around the 446 locus in the 95 *pfkelch* contemporary wild-type infections from the same locations was high (mean H_e_ 0·696, SE 0·001).

**Figure 4:**
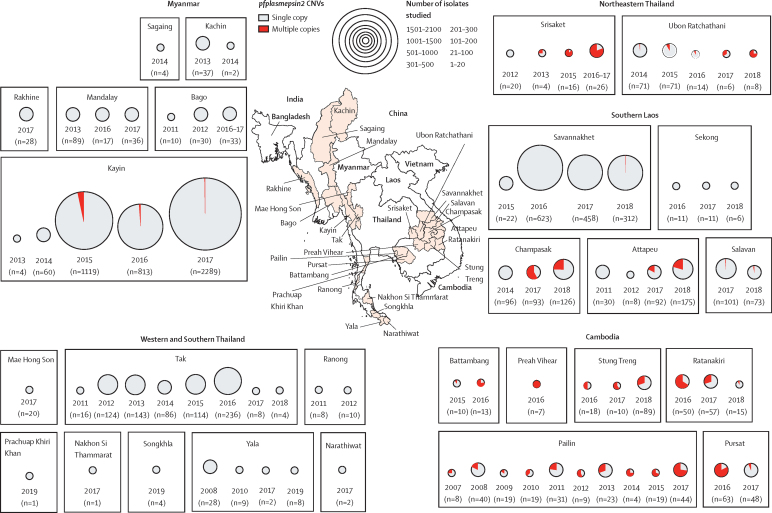
Frequency distributions of *pfplasmepsin2* gene amplification in four countries of the Greater Mekong subregion, 2007–18 CNV=copy number variation.

Between Jan 1, 2007, and Dec 31, 2018, *pfplasmepsin2* amplification was considerably more frequent in the eastern Greater Mekong subregion (n=4571 samples; median copy number 1·83, range 1·60–2·76) than in Myanmar where it was found only in Kyain Seikgyi, Kayin state (2015–17; 51 [1%] of 4221; figure 4). To investigate possible importation from the eastern Greater Mekong subregion, the amplicons and their breakpoint junction sequences were compared. The amplicon containing multiple-copy *pfplasmepsin2* genes in Pailin (west Cambodia), characterised previously, was around 18 kb in length and carried three genes: *pfplasmepsin1, 2*, and *3*.^9^ None of the 36 isolates from Myanmar with amplified *pfplasmepsin2* genes had the same breakpoint as the Cambodian isolates and the amplicons varied in length from 81 kb to more than 190 kb. These findings suggest independent origins for *pfplasmepsin2* amplification in Myanmar and provide no evidence for selection or importation from the eastern Greater Mekong subregion.

Flanking sequences around the *pfplasmepsin2* gene in 51 multiple-copy isolates and 54 single-copy isolates from the eastern Greater Mekong subregion were compared (appendix p 7). There was reduced diversity in the eastern Greater Mekong subregion (mean H_e_ 0·271, SE 0·005), compared with high diversity in wild-type isolates (mean H_e_ 0·608, SE 0·034; p <0·0001; appendix pp 8–9). In Myanmar, there was no evidence of a selective sweep; flanking sequences around multiple-copy *pfplasmepsin2* isolates from Kayin states (n=10) showed different haplotypes (appendix pp 8–9), suggesting different origins (H_e_ 0·643, SE 0·057) and providing no evidence of selection by the intensive use of dihydroartemisinin–piperaquine in mass treatments.

**Figure 5:**
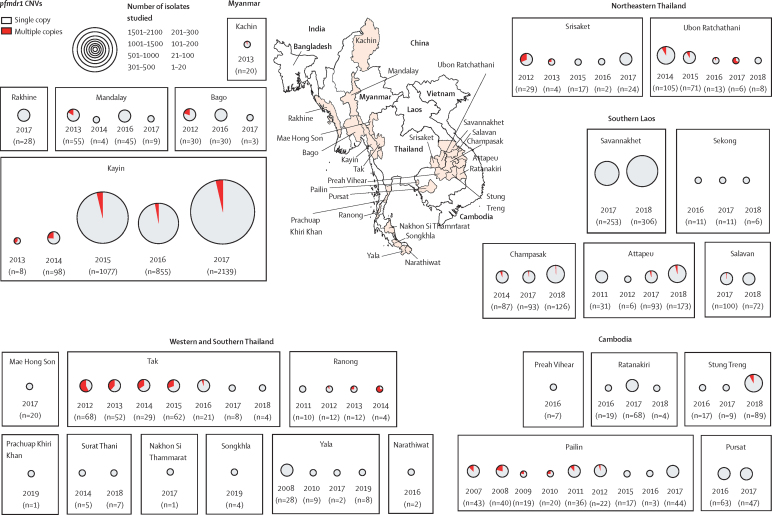
Frequency distributions of *pfmdr1* gene amplification in four countries of the Greater Mekong subregion, 2007–18 CNV=copy number variation.

Between Jan 1, 2007, and Dec 31, 2018, 6984 *P falciparum* isolates were assessed for *pfmdr1* amplification. Amplified *pfmdr1* was found across the region (figure 5); in Mandalay (eight [13%] of 59 in 2013–14) and Kayin, (79 [3%] of 2139 in 2017) in Myanmar and on the northwestern border of Thailand in Mae Sot (19 [30%] of 62 in 2015). This figure for Mae Sot is slightly lower than the proportion in 1993–94 in the same area (30 [48%] of 62) when mefloquine monotherapy was first-line treatment.^4^, ^12^ *pfmdr1* amplification was also found in southern Laos (seven [4%] of 173 in 2018), and Stung Treng, northern Cambodia (seven [8%] of 89 in 2018), and in northeastern Thailand (Ubon Ratchathani and Srisaket) near the Cambodian border (one [7%] of 13 in 2016).

**Figure 6:**
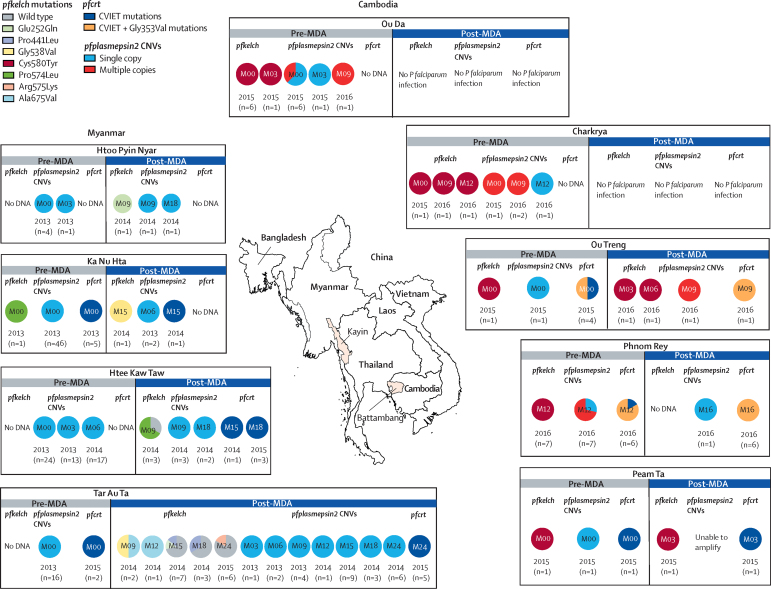
Frequency distributions of *pfplasmepsin2* gene amplification, *pfcrt*, and *pfkelch* gene mutations in *Plasmodium falciparum* isolates from submicroscopic parasitaemias in eastern Myanmar and western Cambodia, before (n=142) and after (n=38) mass drug administration with dihydroartemisinin and piperaquine M inside the circles refers to the month of study. Areas the isolates were collected from are shown on the map in pink. CNV=copy-number variation.

With regard to submicroscopic *P falciparum* parasitaemias, the proportion of isolates with *pfplasmepsin2* amplification was low or zero before (n=142) and after (n=38) mass treatment with dihydroartemisinin–piperaquine in the sites in Myanmar and Cambodia. There was also no evidence for selection of *pfcrt* mutations associated with piperaquine resistance; none were found in Kayin State, Myanmar, and *pfcrt* Gly353Val was found in seven isolates before and seven after mass treatment in Cambodia (with a single haplotype of CVIET at residues 72–76; figure 6; appendix p 15).^10^, ^11^, ^12^, ^13^, ^27^ There was also no evidence for further selection of *pfkelch* mutations after mass treatment.

## Discussion

Antimalarial drug resistance in *P falciparum* continues to evolve in the Greater Mekong subregion, making the treatment of falciparum malaria increasingly difficult and threatening national and regional malaria elimination targets. Artemisinin resistance, first recognised in 2007 in western Cambodia, is closely associated with mutations in the *pfkelch* gene (chromosome 13) propeller region.^3^, ^7^ Initially multiple independent emergences of artemisinin resistance occurred,^14^, ^27^, ^28^ but hard selective sweeps by presumably fitter parasites have gradually replaced the first soft selective sweeps.^15^, ^24^ The reduced parasite killing associated with artemisinin resistance increased the selective pressure on their ACT partner drugs, and resistance to them followed. ACT treatment efficacy has declined correspondingly.^3^, ^4^, ^5^, ^6^

In terms of malaria epidemiology, the Greater Mekong subregion can be divided into two discrete transmission regions: the eastern region, comprising Cambodia and adjacent southern Vietnam, southern Laos, and north-eastern Thailand, and the western region, comprising Myanmar and adjacent western Thailand, with borders with Yunnan province, China, northeast India, and Bangladesh. The area between these two regions, comprising northern Laos and the main body of Thailand, is largely malaria free. In the eastern region, except for Laos, dihydroartemisinin–piperaquine has been used extensively for many years. A single long haplotype *pfkelch* Cys580Tyr mutant parasite lineage (PfPailin) has now spread across the eastern Greater Mekong subregion and has also acquired piperaquine resistance (associated with *pfplasmepsin2* amplification and *pfcrt* mutations).^15^, ^24^ Dihydroartemisinin–piperaquine was initially highly effective and became first-line treatment in Vietnam, Cambodia, and Thailand, but as the multidrug-resistant PfPailin lineage came to dominate in the eastern Greater Mekong subregion, high rates of treatment failure occurred. Treatment failure increased with the acquisition of mutations in *pfcrt*,^11^ which reduced piperaquine susceptibility,^10^, ^25^ and *pfplasmepsin* amplification, which is associated with reduced piperaquine susceptibility. These new *pfcrt* mutations are downstream of the 4-aminoquinoline resistance locus (positions 72 to 76), which is mutated as the CVIET haplotype in nearly all *P falciparum* parasites from the region.

The majority of falciparum malaria in Thailand used to occur in areas adjacent to Myanmar, with the highest case numbers in Tak province.^29^ Since provision of support for village malaria workers and introduction of concerted malaria elimination activities (including mass drug administrations) in adjacent Kayin state in eastern Myanmar,^17^, ^30^, ^31^, ^32^ the incidence and prevalence of falciparum malaria on both sides of the border has dramatically declined.^29^ Before these large-scale interventions, a single *pfkelch* Cys580Tyr haplotype predominated.^27^ Furthermore, despite intense use of dihydroartemisinin–piperaquine in treatment and in mass treatments in Kayin state^17^, ^31^ there is no evidence for emerging piperaquine resistance there.^33^ No *pfplasmepsin2* amplification and no *pfcrt* mutations associated with piperaquine resistance were identified in this region and, although low prevalences of *pfplasmepsin2* amplification were found elsewhere in Myanmar, there was no evidence for selection. There was also no evidence for the spread of drug-resistant *P falciparum* from the east to the west of the Greater Mekong subregion. There is therefore still a window of opportunity to continue the elimination of multi-drug resistant malaria in the western Greater Mekong subregion using dihydroartemisinin–piperaquine in targeted mass treatments, as proved successful in eastern Kayin state. In Myanmar, a *P falciparum* lineage bearing the *pfkelch* Phe446Ile mutation,^16^, ^18^ recognised first on the northern border with Yunnan, China, has now spread extensively across the country. In northern Myanmar, Phe446Ile comprises the majority of *pfkelch* propeller mutants. This evolving pattern, in which a single parasite lineage dominates, is similar to that observed earlier in the eastern Greater Mekong subregion with the Cys580Tyr lineage (PfPailin).^15^ The *pfkelch* Phe446Ile mutation confers a lesser degree of artemisinin resistance (in terms of slowing of parasite clearance) than Cys580Tyr,^3^, ^18^ which has not dominated in areas other than those close to the Thai border. In areas of higher malaria transmission in remote regions of Myanmar, the putative lesser fitness disadvantage conferred by the Phe446Ile mutation might be relatively more advantageous in the context of ongoing competition from wild-type parasites and continued selection by treatments containing artemisinin.

The development and spread of piperaquine resistance in the eastern Greater Mekong subregion has raised concerns that resistance could spread further afield or emerge elsewhere. In this study, *pfplasmepsin* amplification was identified in some *P falciparum* isolates from Myanmar but the mechanism of amplification differed to that in eastern Greater Mekong, and the sequences flanking the amplified genes were unrelated. Whether these strains are piperaquine resistant in vivo is unknown, but the diversity of haplotypes does suggest that they are not being selected. Piperaquine has not been used in these areas. In Kayin state and adjacent Thailand, where dihydroartemisinin–piperaquine has been used extensively in mass treatments,^17^ analysis of *P falciparum* parasites in or around the elimination areas shows no evidence that mass treatments selected for artemisinin or piperaquine resistance. This finding supports the use of targeted mass treatment in a low transmission setting to reduce the burden of malaria in foci of high transmission, thereby reducing the risk of resistance emerging.^33^

*pfmdr1* amplification occurs readily within malaria infections and was largely responsible for the rapid reduction in mefloquine susceptibility on the eastern and western borders of Thailand in the early 1990s before ACTs were introduced.^12^ Despite this reduction in mefloquine susceptibility, artesunate–mefloquine proved highly effective in the same areas and gave sustained efficacy for more than 15 years before failure rates increased sharply again in 2011.^34^ The reduction in artesunate-mefloquine efficacy resulted from the combination of artemisinin resistance (associated with *pfkelch* mutations) and re-emerging mefloquine resistance (associated with *pfmdr1* amplification).^4^ Our survey shows that the prevalence of *pfmdr1* amplification has declined again on both the eastern and western borders of Thailand and adjacent countries after artesunate-mefloquine was replaced as first-line treatment. This finding is somewhat reassuring for artemether-lumefantrine, the first-line treatment in Myanmar^19^ and Laos, although how much longer efficacy will be preserved in the face of artemisinin resistance is uncertain. Concern over the longevity of current ACTs has led to proposals for use of triple ACTs to protect the more slowly eliminated antimalarial drugs and prolong their utility.^11^

The main limitation of this study is that sampling was usually associated with studied interventions, so it was focused in areas already known to have a higher incidence of malaria than elsewhere. Although the tested samples were obtained from multiple sites across the region this was not planned as a geographic survey and, as with many other observational molecular epidemiology studies, might therefore give a biased picture of genotype distributions. Surveillance by genotyping routine *P falciparum* positive rapid diagnostic test samples from village health workers would clarify the geographic distribution and monitor the spread of resistance. The marked reduction in the incidence of falciparum malaria in areas where targeted malaria elimination activities have been done means that few parasite isolates remain to provide accurate estimates of the prevalence of resistance markers after the interventions. But the low number is itself a testament to success. Nevertheless, the overall size, duration, and geographic extent of this investigation does allow characterisation of the emergence and spread of antimalarial drug resistance in the region, and it gives confidence in concluding that dihydroartemisinin–piperaquine mass treatments have not selected resistance further.

The WHO strategy for malaria elimination in the Greater Mekong subregion (2015–30)^35^ set the following targets: by 2020 or earlier transmission of *P falciparum* malaria to be interrupted in all areas of multidrug resistance, including ACT resistance; by 2020 *P falciparum* malaria to be eliminated in Cambodia; and by 2025 *P falciparum* malaria to be eliminated in all Greater Mekong countries. These targets will clearly not be met. The selection and subsequent spread of fit multidrug-resistant parasites across the large landmasses of southeast Asia has been repeatedly highlighted as a major threat to global malaria control. The solution is to eliminate falciparum malaria in the Greater Mekong subregion before it spreads further. There is still time for radical action before drug resistance prevents it.

## Data sharing

The data are available upon request to the Mahidol–Oxford Tropical Medicine Research Unit Data Access Committee for researchers and access follows the Mahidol–Oxford Tropical Medicine Research Unit data access policy. Queries and applications for datasets should be directed to Rita Chanviriyavuth (Mahidol–Oxford Tropical Medicine Research Unit; rita@tropmedres.ac).
