# Supplementary material for: Molecular epidemiology of resistance to antimalarial drugs in the Greater Mekong subregion: an observational study
Source: Lancet Infect Dis. 2020 Dec;20(12):1470–80. doi: 10.1016/S1473-3099(20)30228-0 (PMC7689289; doi:10.1016/S1473-3099(20)30228-0)
Supplement: Supplementary appendix [file mmc1.pdf]

THE LANCET
Infectious Diseases

Supplementary appendix

This appendix formed part of the original submission and has been peer reviewed. We post it as supplied by the authors.

Supplement to: Imwong M, Dhorda M, Tun K M, et al. Molecular epidemiology of resistance to antimalarial drugs in the Greater Mekong subregion: an observational study. *Lancet Infect Dis* 2020; published online July 14. https://doi.org/10.1016/S1473-3099(20)30228-0.

Molecular epidemiology of resistance to current antimalarial drugs in the Greater Mekong Subregion: an observational study

Supplementary material

S1 figure.

Number of samples and specimen types analyzed in this study

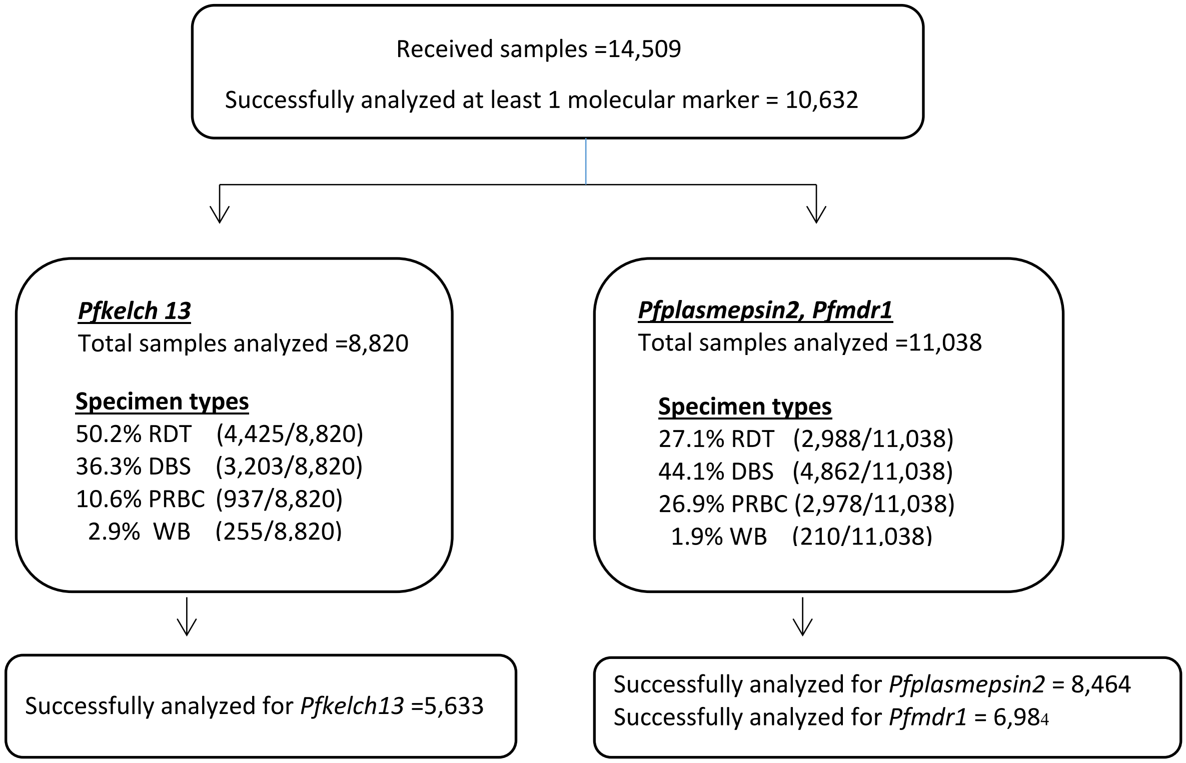

RDT: malaria rapid diagnostic test, DBS: dried blood spot, PRBC: packed red blood cells, WB: whole blood..

S2 figure.

Positions of fifteen microsatellite markers adjacent to *pfplasmepsin2/3* on chromosome 14

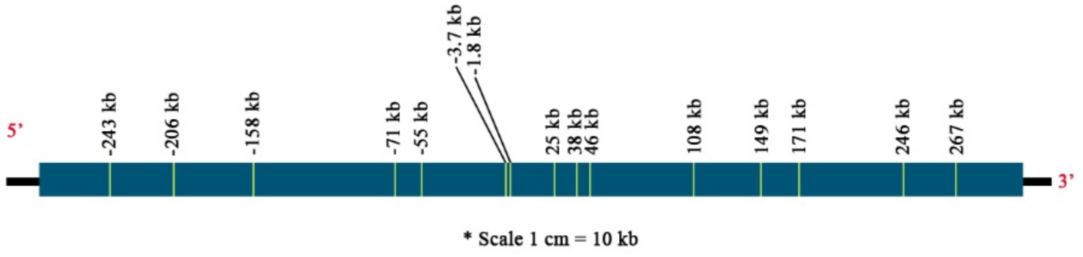

S3 figure.

Positions of real time PCR primers adjacent to *pfplasmepsin2/3* (red) on chromosome 14

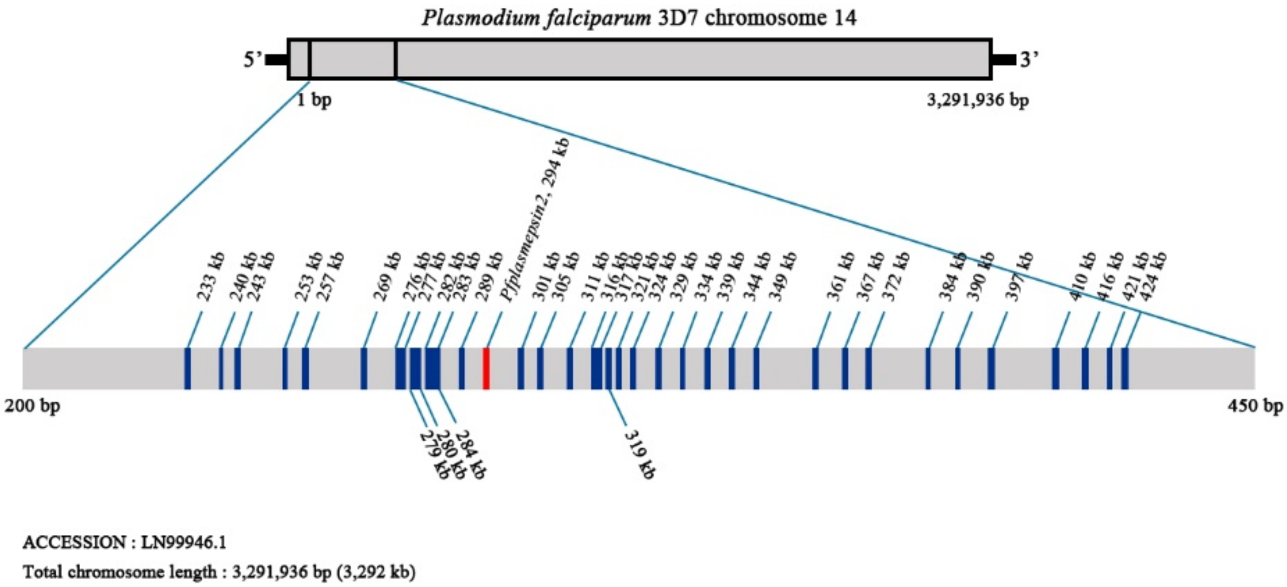

S4 figure.

A) Reduced diversity in 14 microsatellite markers surrounding the *pfkelch C580Y* allele in parasites from Myanmar from 2014-2017 (green) and in the subgroup of these obtained between 2015 and 2017 (red) compared to wild type *pfkelch* from the same study sites (grey line). Arrow marks the gene locus.

B) Mean (SE) heterozygosity (H_e) of wild type and mutant alleles in parasite samples from Myanmar 2014-2017. * are the p- values for the comparisons with the He of wild types at each site. ** are the p- values for the comparisons with the He of Myanmar 2014 for each site. The overall chi-square for trend is 50.3, $p<0.0001$ with 1 degree of freedom for comparing the proportions (0.367, 0.266, 0.216, 0.235) over time i.e. 2014, 2015, 2016 and 2017 respectively.

A)

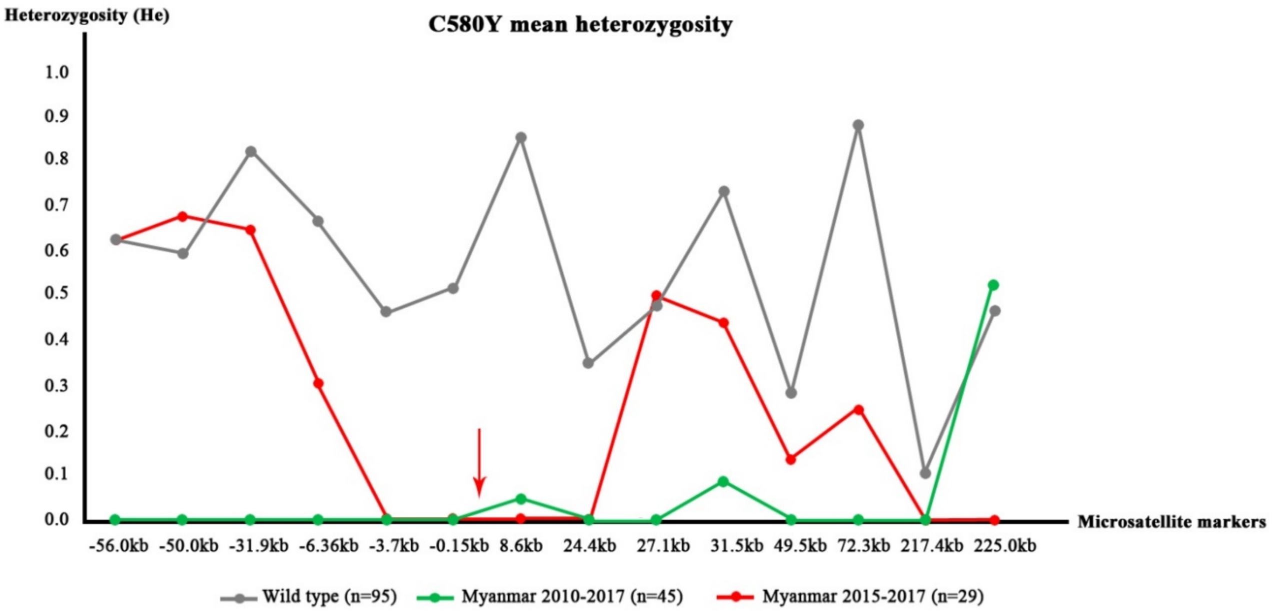

B)

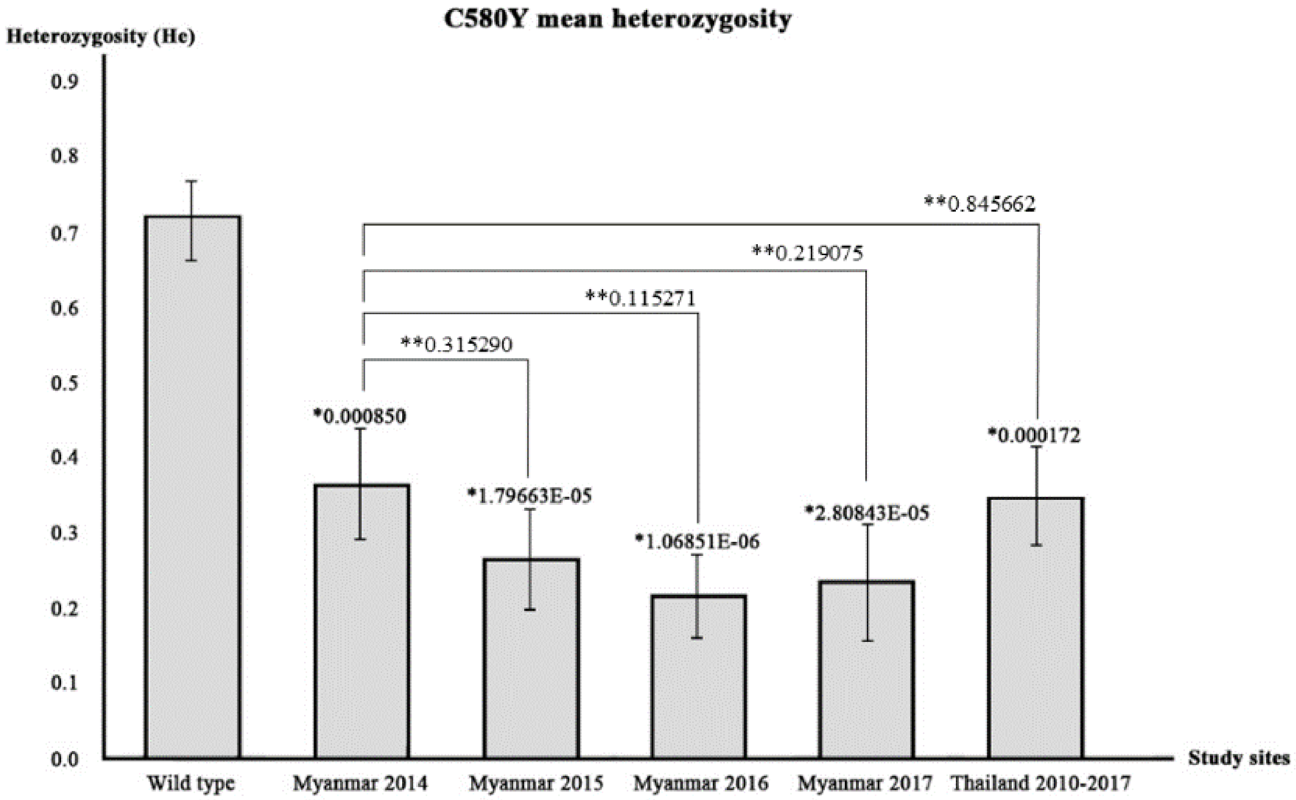
C580Y mean heterozygosity
Heterozygosity (He)
0.9
0.8
0.7
0.6
0.5
0.4
0.3
0.2
0.1
0.0
**0.845662
**0.219075
**0.115271
**0.315290
*0.000850
*1.79663E-05
*1.06851E-06
*2.80843E-05
*0.000172
Study sites
Wild type
Myanmar 2014
Myanmar 2015
Myanmar 2016
Myanmar 2017
Thailand 2010-2017

S5 figure.

A) Reduced diversity in 14 microsatellite markers surrounding the *pfkelch* F446I allele in parasites from Myanmar from 2014-2017 (red) compared to wild type *pfkelch* from the same study sites (grey line). Arrow marks the gene locus.

B) Mean (SE) heterozygosity (H_e) of wild type and F446I mutant alleles in parasite samples from Myanmar. * are the p- values for the comparisons with the He of wild types at each site. ** are the p- values from the comparisons with the He of Myanmar 2014 for each site.

A)

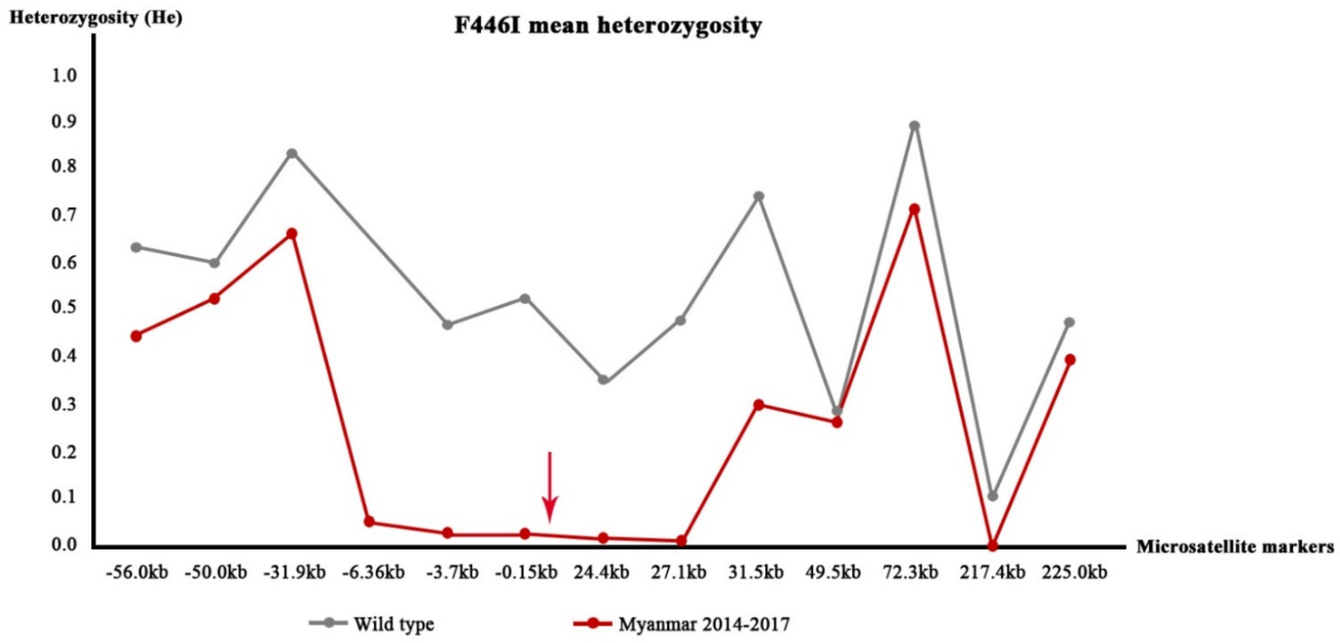

B)

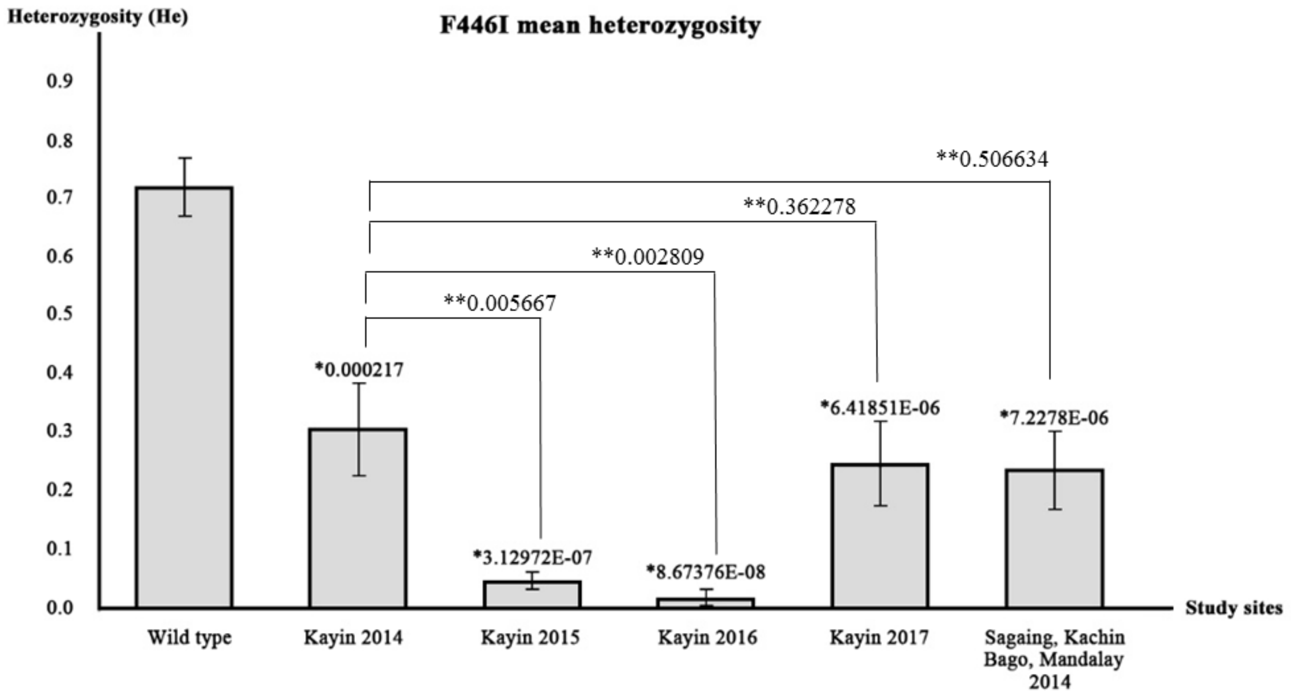

F446I mean heterozygosity
Heterozygosity (He)
0.9
0.8
0.7
0.6
0.5
0.4
0.3
0.2
0.1
0.0
**0.506634
**0.362278
**0.002809
**0.005667
*0.000217
*3.12972E-07
*8.67376E-08
*6.41851E-06
*7.2278E-06
Study sites
Wild type
Kayin 2014
Kayin 2015
Kayin 2016
Kayin 2017
Sagaing, Kachin Bago, Mandalay 2014

S6 figure.

Polyallelic data at the plasmepsin2/3 gene and in a -243 to +246kb interval surrounding it (chromosome 11). Data are shown for the 10 *pfplasmepsin2* amplified isolates from Kayin state, Myanmar (bottom section) and 95 isolates from Cambodia. Position 0 on the x-axis is the *pfplasmepsin2,* whereby red is amplified and green is wild type. As for Figures 2 & 3, each marker has an independent colour schema. The total number of distinct alleles observed in all the data for each marker is given by the number in parentheses.

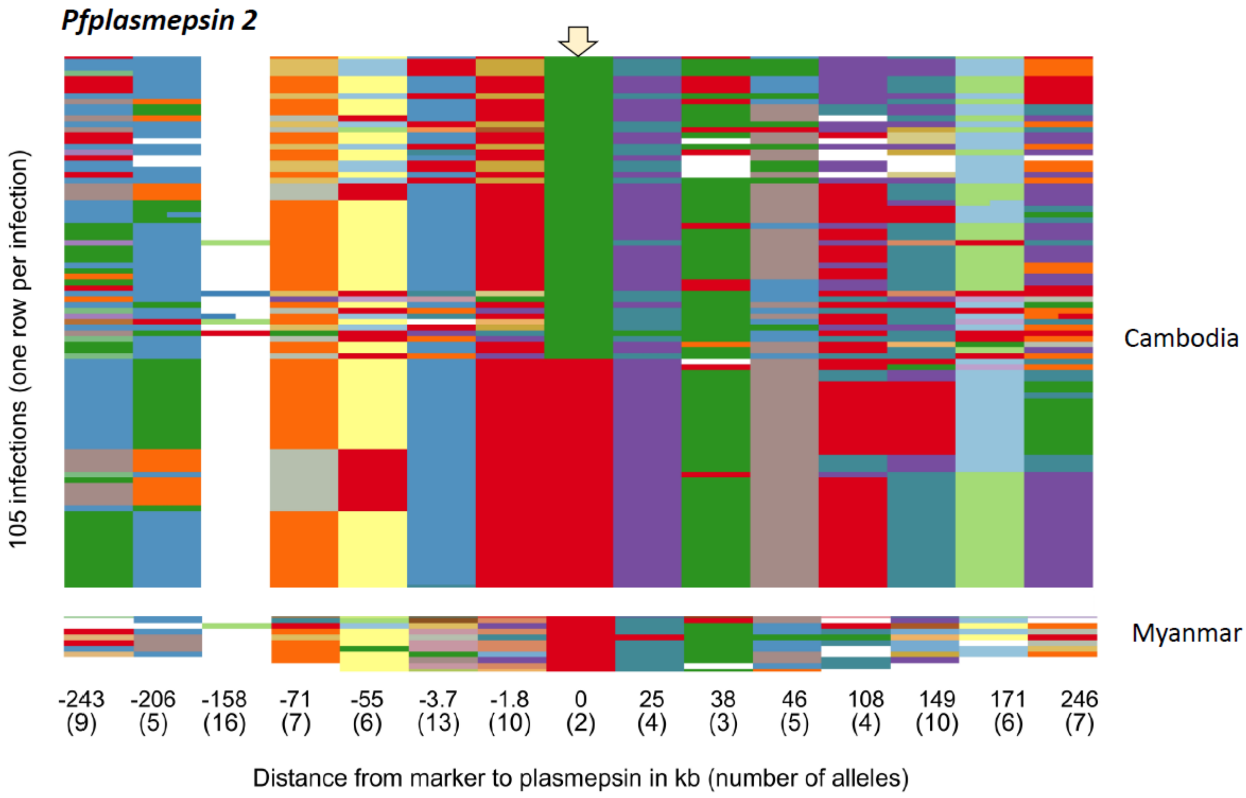

S7 figure.

A) Reduced diversity in 15 microsatellite markers surrounding the multiple copy- *pfplasmepsin2* allele in parasites from Ubon Ratchathani Thailand (green line), Pailin Cambodia, from 2008 to 2017 (red line) and the ten isolates from Myanmar with multiple copy *pfplasmepsin2* isolated from 2015 to 2017 (blue line) all compared to wild type *pfplasmepsin2* from the same study sites (grey line).

B) Mean (SE) heterozygosity (H_e) of wild type and multiple copy *pfplasmepsin2* alleles in parasite samples from Pailin, Ubon Ratchathani, and Myanmar. * are the p-values for the comparisons with the He of wild types at each site.

A)

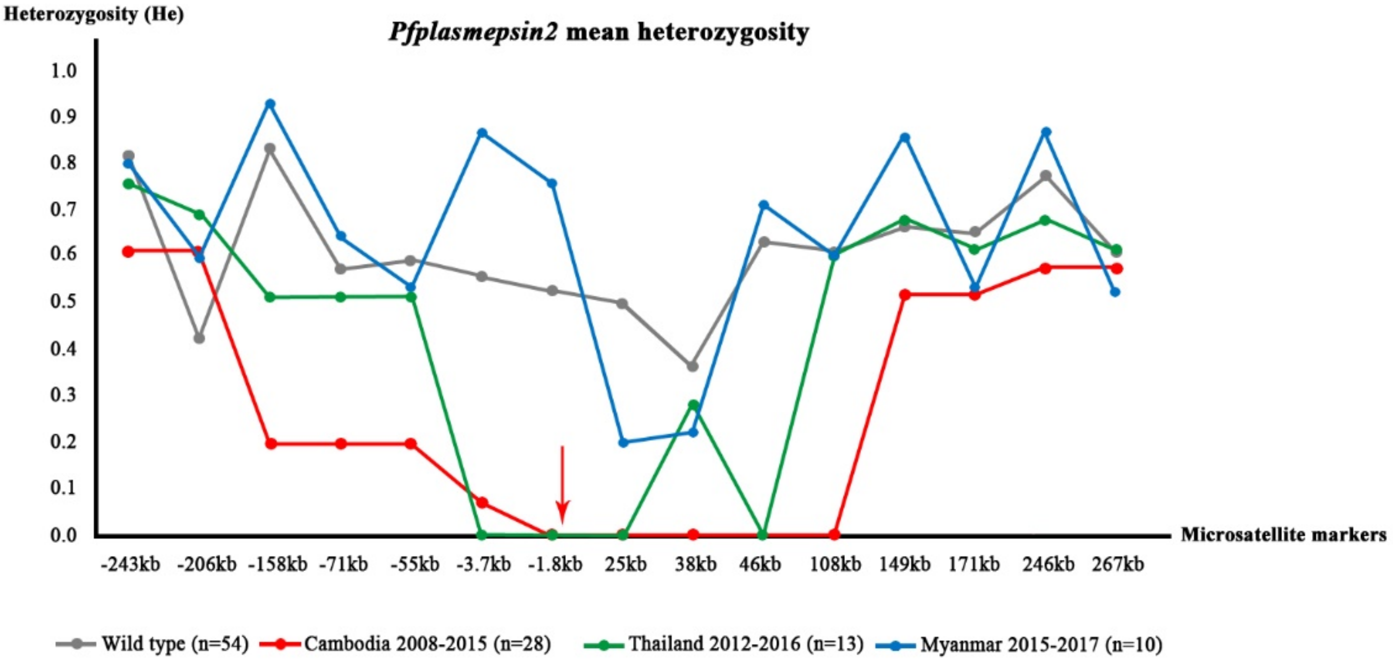

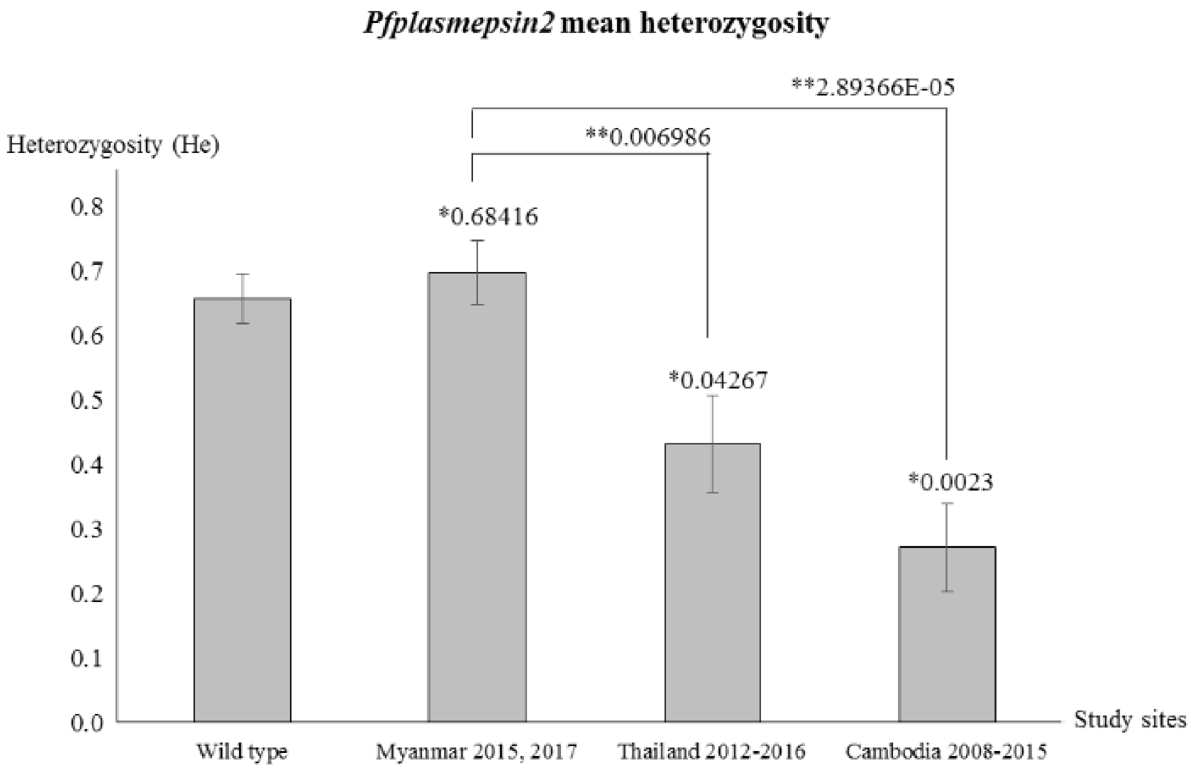

Pfplasmepsin2 mean heterozygosity
Heterozygosity (He)
**2.89366E-05
**0.006986
*0.68416
*0.04267
*0.0023
0.8
0.7
0.6
0.5
0.4
0.3
0.2
0.1
0.0
Study sites
Wild type
Myanmar 2015, 2017
Thailand 2012-2016
Cambodia 2008-2015

S1 table

The numbers of samples from each country

Countries	Study sites	Year	Number of samples
Cambodia	Battambang	2015-2016	64
	Pailin	2007-2017	311
	Preah Vihear	2015	8
	Pursat	2016-2017	125
	Ratanakiri	2016-2018	130
	Steung Treng	2018	130
	**Total**		**768**
Thailand	Mae Hong Son	2017-2018	37
	Nakhon Si Thammarat	2019	1
	Prachuap Khiri Khan	2019	1
	Prachuap Khiri Khan	2019	1
	Ranong	2011-2014	59
	Songkla	2019	10
	Srisaket	2015-2017	50
	Tak	2011-2018	804
	Ubon Ratchathani	2014-2018	243
	Yala	2008-2019	167
	**Total**		**1373**
Lao PDR	Attapeu	2011-2012, 2017-2018	377
	Champasak	2014-2018	350
	Salavan	2017-2018	196
	Savannakhet	2017-2018	1923
	Sekong	2017-2018	32
	**Total**		**2878**
Myanmar	Bago	2011-2012, 2016-2017	113
	Chin	2014	58
	Kachin	2011-2014	245
	Kayin	2013-2017	8254
	Mandalay	2011-2017	225
	Rakhine	2013-2014, 2015-2017	430
	Sagaing	2013-2015	115
	Shan	2013-2014	50
	**Total**		**9490**
**Total**			**14509**

Full details of these clinical and epidemiological studies (clinical trials.gov: NCT01350856 and NCT02020330) have been published previously. Samples were also obtained as part of studies on the epidemiology, treatment and the targeted elimination of artemisinin resistant malaria (Trial registration

numbers NCT01350856, NCT02453308, NCT03384498, NCT01872702, and NCT01872702) conducted between 2007 and 2018. Approvals for these studies were obtained from the Ethics Review Boards of the Research Ethics Committee of the Department of Disease Control, Thailand Ministry of Public Health (8/57-676), Faculty of Tropical Medicine, Mahidol University; TMEC 17-030**,** TMEC 11-003 (MUTM 2011-015-01), Faculty of Medicine, Prince of Songkla University, Thailand (REC60-096-19-2); Department of Medical Research, Ministry of Health Myanmar; TRAC1 DMR UM and TRAC1 DMR LM, DMR Lower Myanmar Letters No.50/Ethics 2013, No.73/Ethics 2014, and Ethics/DMR/2015/109E and 113E, DSMA Letter No.03/Ethics/13. The Defence Services Medical Ethics Committee (Myanmar) DSMRC No.1/2/IRB-7, DSMRC IRB/2015/39, IRB/2016/12 and IRB/2017/86; the Ministry of Health in Cambodia, and the University of Oxford Tropical Medicine Ethics Committee (OxTREC Protocol Numbers: 527-17, 06-11, 06-15, 5106-16, 1017-13, 1015-13).

S2 table.

Primer sequences of 15 microsatellite markers adjacent to *Pfplasmepsin2/3*

NO.	Primer name	Product size	Consensus pattern	LEFT PRIMER	RIGHT PRIMER
1	-243KB	300	AT	FAM-ATTGTTTGCCCCTATACCATCTTTA	AATGTTCCTGTGATAAGGATTTGG
2	-206KB	227	TAT	FAM-TTAAGACCAAAATTGCCTACAGC	GATACAAAGGCGAAGACGTTAAA
3	-158KB	268	AT	FAM-CCAAAAACAAAAAGAAACACAA	TTAGAAAGACGCAAGATATTTTCA
4	-71KB	281	AAC	FAM-TGAAGAAGGTATGTTGAAAATGG	TGTCCTTGTTATTATGGTCCTTG
5	-55KB	259	ATA	FAM-TGGAATGTAAAACCCCATTTGTA	ATATTCGCTCCTTTTGTACATGC
6	-3.7KB	300	AT	FAM-CCTGTTGATTTGAACAAAAATACC	AGCTTTAGCATCATTCACGTTTTA
7	-1.8KB	291	TA	FAM-GAATTATTTGATATTGATTTTCATGTC	AAGTCAGCTAGTTCCTTATTCTTTCA
8	25KB	298	TAA	FAM-AAGGAAAGCAATAAAGCTACAAGC	TTTCTAAATCCTTACCTGCTTTCG
9	38KB	217	TAT	FAM-GAATAGAGCTCATGGAACCATTCT	AAACAAAAGGCCAAAACACATATT
10	46KB	258	TAT	FAM-GAATTGATGGACTCTGTGTCATGT	GATGACAGGTAAGGAAGAGCAAAT
11	108KB	292	AAAT	FAM-TGTCAGAATGGAAATTTGTGAAA	CGTTTTGGTATCAAGACAAAATGA
12	149KB	300	ATA	FAM-ACGTATTTAAAGGGATGTGAACAAA	AAAGGGCCTATTACTTAACAGTTCT
13	171KB	283	TTG	FAM-TCTTGACTTATTGTCATGGCATC	AAACGAATGGAATCAACAGTAACA
14	246KB	221	AT	FAM-TGGGGCTTATTATTTATGTGCTC	TTCAATGACGAAAAGGAAAAGAA
15	267KB	295	AGGAGC	FAM-GAAAGACACATCAAACATATCTTCA	TTCAACAAATGTATTGGCAGATG

All primary PCR reactions were carried out in a total volume of 20µL. All reactions contained 10 mM Tris-HCl, pH 8.3, 50 mM KCl , 2 mM $MgCl_2$, 125 µM dNTPs, 250 nM of each primers in the primary reaction and secondary reaction, respectively, and 0.4 units of Taq Polymerase (Invitrogen, USA). Primer sequences are shown in the table. In the PCR reactions 2 µL of genomic DNA was the template. PCR was performed using the following cycling parameters: an initial denaturation step at 95°C for 5 minutes, followed by 35 cycles of denaturing at 94°C for 1 minute, annealing at 55°C for 2 minutes and extension at 72°C for 2 minutes, followed by a final extension step at 72°C for 5 minutes. Internal size standards (Genescan® 500 LIZ) were used to measure the length of the PCR generated products and Genescan® and Genotyper® software (PE Applied Biosystems) was used to measure allele lengths and to quantify peak heights.

S3 table. Primer sequences of 37 real time PCRs adjacent to *Pfplasmepsin2/3*

No.	Primers	Primer name	Sequences (5'->3')
1	Forward	PF14_233kb_F	TCCTTTTTCCTCTCCTTACTTTCA
	Reverse	PF14_233kb_R	TTAAATCCGAATGTCATGAAGC
2	Forward	PF14_240kb_F	TTTATGAGCAGTCAGGAAGTGC
	Reverse	PF14_240kb_R	CCATTTTCGTTTTGTTTTCGAT
3	Forward	PF14_243kb-F	TTGGTGCATACTTCTAATGGAAA
	Reverse	PF14_243kb-R	TGCCGTGTGTGTATGATTGA
4	Forward	PF14_253kb-F	TGGTCAATTATATGCTGGTGAGA
	Reverse	PF14_253kb-R	TTTTCGGTTCATAATCTACAAAAGG
5	Forward	PF14_257kb-F	TGCATAGAAAATTGTACAAAATCAAAA
	Reverse	PF14_257kb-R	TCCTGCATGTATTGCTGCTT
6	Forward	PF14_269kb-F	CTTCTTTTTCCATTTTCTTCCAAGT
	Reverse	PF14_269kb-R	AAATTTTTACGACTATCAAGCGAGTT
7	Forward	PF14_276kb-F	TTCAAGTCCTTTTTATTTTTCTCCAT
	Reverse	PF14_276kb-R	AACAACAAAACGGTGGAAAGA
8	Forward	PF14_277kb-F	TGAATATTCTTTCATTTTGGTTGTTT
	Reverse	PF14_277kb-R	TTGATGTAGTTTTCCTCTGTTCCA
9	Forward	PF14_279kb-F	TAGCCATAACCATAATGTTGTTAAA
	Reverse	PF14_279kb-R	TTATGAATTGTGATACCTTACCTGA
10	Forward	PF14_280kb-F	ATGGACATTTATTGAAACAAAAG
	Reverse	PF14_280kb-R	GCAAGCCAAATCTTGAGACA
11	Forward	PF14_282kb-F	TTCTCTCTTTTTCTAACGTAACTTTTC
	Reverse	PF14_282kb-R	TGCGCTTAAATAGATCCTTCA
12	Forward	PF14_283kb-F	TGAAGAACTTGAAAATCCAGAAA
	Reverse	PF14_283kb-R	TGCGATGTGAATAAAAATTTCC
13	Forward	PF14_284kb-F	CCATTATATGTAACCACCTGTGATA
	Reverse	PF14_284kb-R	GGTCCATATAGAATTCAGGTTCTAA
14	Forward	PF14_289kb-F	TTAACTGTAGAAAAAGCTACTGCAA
	Reverse	PF14_289kb-R	AACAACATCTAAACCTTCAAAGAAT
15	Forward	PF14_301kb-F	GCTCTTCTTTTACCACCACTAACT
	Reverse	PF14_301kb-R	CAGGAGTGTCAGGAGTATATTTTG
16	Forward	PF14_305kb-F	ATTCGTATGGGTTAGATTCTTGTA
	Reverse	PF14_305kb-R	ATATTTGTTAAATGCAGGAGATGA
17	Forward	PF14_311kb-F	GTGTAATAAGCAAATGATGGAAAT
	Reverse	PF14_311kb-R	TATGTCATTTTATTTTCTTCACAGAT
18	Forward	PF14_316kb-F	AAAATATTAAGACCCCATAAGGAAT
	Reverse	PF14_316kb-R	TTATTATCCACATATGAAAATCGAG
19	Forward	PF14_317kb-F	TCTCCATTTTTCCTCGTACGTT
	Reverse	PF14_317kb-R	AGGTTCTCGAGATCAATTATGTG
20	Forward	PF14_319kb-F	TGAACACATTAAAATTAACAACATGA
	Reverse	PF14_319kb-R	CAAAAATTATGTCCCTCGCATA
21	Forward	PF14_321kb-F	AACAAACCTATAAGAAGAACGTACAA
	Reverse	PF14_321kb-R	TATTTTCTAAATCCTTACCTGCTTT
22	Forward	PF14_324kb-F	CACATTCGGAACAACCCTTG
	Reverse	PF14_324kb-R	TCCTTTGCAGCTTCTTCGTT
23	Forward	PF14_329kb-F	TTGTTCCTCTTCCCCATGAC

	Reverse	PF14_329kb-R	CACGATACAAACGATGACAAAAA
24	Forward	PF14_334kb-F	CTTTGTCATTTCCTTCATCTTGATT
	Reverse	PF14_334kb-R	CAATGGAATCAACCACCACA
25	Forward	PF14_339kb-F	TGGAAGGAACAAAAGTTGACA
	Reverse	PF14_339kb-R	AGTGAAGGGTATGAAAAATATCATGT
26	Forward	PF14_344kb-F	TCCGCCTCTTTTGGTTCATA
	Reverse	PF14_344kb-R	TGATTTCTTGTTACGAGTTAAATGTGT
27	Forward	PF14_349kb-F	AAAATCCAACAGCACAATATTAAA
	Reverse	PF14_349kb-R	TGCTTGGTCATATTGCTTTTT
28	Forward	PF14_361kb-F	TTCTCATCATCCTCATCGTCA
	Reverse	PF14_361kb-R	TAATGTTGGAAATAAGATAAATAAAA
29	Forward	PF14_367kb-F	CCCCCCTTTTCAACCTTC
	Reverse	PF14_367kb-R	TTCTTCTTCGACAGAATATCAAGG
30	Forward	PF14_372kb-F	TCCTCATATTTTGCACATCCA
	Reverse	PF14_372kb-R	CCCGCATGGTTCTTCTTTAT
31	Forward	PF14_384kb-F	CCTTAGGGCCATTGATTGTT
	Reverse	PF14_384kb-R	TTCATTAGCATTATTCATATTCACACC
32	Forward	PF14_390kb-F	AATGACAAATAAATGCGCAAA
	Reverse	PF14_390kb-R	CGGTTGAGCTCTCAGTACTCTTC
33	Forward	PF14_397kb-F	ATATATAAACATTTATTTTCGAGCACA
	Reverse	PF14_397kb-R	TAGCCCGGCTGCACTTATTA
34	Forward	PF14_410kb-F	CTTGGTTCACATAACCCCTCA
	Reverse	PF14_410kb-R	TTGTCTGATTGTGCCGATAAA
35	Forward	PF14_416kb-F	TTCCTCCTCTTCACTCTCAAGAC
	Reverse	PF14_416kb-R	TGAGTGAAACACAAAGGGAAAA
36	Forward	PF14_421kb-F	GGAAAAGAATCAGACGAAGAACC
	Reverse	PF14_421kb-R	AATTTACTGAAAAATCGAGAAAAGG
37	Forward	PF14_424kb-F	AGATGCTTTTTGTTTTGCTTTT
	Reverse	PF14_424kb-R	CCCAGATTGTCGATCAGTGAG
38	Forward	PF-B-tubulin-F	AAAAATATGATGTGCGCAAGTGA
	Reverse	PF-B-tubulin-R	AACTTCCTTTGTGGACATTCTTCCT

The copy number variations of the *P. falciparum plaspmepsin 2* gene flanking regions were measured using the SYBR green relative quantitative real-time PCR assay QuantStudio 7 Flex Real-Time PCR System (Thermo Fisher Scientific, Waltham, MA, *USA*). The singleplex real-time PCR reaction was prepared in 20 ul, containing 10 ul of 2X SsoFast EvaGreen® (BioRad), 1 ul of 10µM forward and reverse primers mix, 7 ul of nuclease free water, and 2 ul of DNA samples. In each set of reactions. The *P. falciparum* 3D7 strain (single copy) was used as a calibrator (in triplicate). A negative control (reagents only) was also performed each time. There were three stages in the run method using QuantStudio^{tm} Flex System including a hold stage (1 cycle of 98°C for 2 minutes), PCR stage (45 cycles of 98°C for 5 seconds, 60°C for 30 seconds with data collection), melt curve stage (1 cycle of 95°C for 15 seconds, 60°C for 60 seconds with data collection every 5 seconds, and 95°C for 15 seconds with data collection). The copy number of the flanking region was calculated by the formula = $2^{\Delta\Delta Ct}$, where ΔΔCt is calculated from the threshold cycle (Ct) of samples using the formula: ΔΔCt = (Ct *pf* flanking region − Ct *pf β-tubulin*) of sample − (Ct *pf* flanking region − Ct *pf β-tubulin*) of *P. falciparum* 3D7. A cut-off copy number of 1.5 was used to define amplification at the flanking regions.

S4 table. Oligonucleotide primers used for amplifying across unique junctions of multiple copy-*Pfplasmepsin2/3*

**Name**	**Sequence 5'->3'**	**Length**	**Tm**	**%GC**
Pm1_1359R	AATTTTTTTTTGGCAAGGGC	20	58.92	35
Pm3_982F	GTATCTTCCAAAAAAGCAAACGTTA	25	58.71	32
Pm1_1548R	GCTTTAGCATCATTCACGTTTTATAA	26	58.74	31

All primary PCR reactions and secondary reactions were carried out in total volume of 20µL except for the secondary reactions for direct DNA sequencing (100µL). All reactions contain 10 mM Tris-HCl, pH 8.3, 50 mM KCl , 2 mM $MgCl_2$, 125 µM dNTPs, 250 nM of each primer in the primary reaction and secondary reaction and 0.4 units of Taq Polymerase (Invitrogen, USA). Primer sequences are shown in the table. In primary reactions 1.5µL of genomic DNA was the template. Then 2 µl of the products of the primary reaction was used as template in the secondary PCR reaction. PCR was performed using the cycling parameters as follows: an initial denaturation step at 95°C for 5 minutes, followed by 25 cycles for the primary PCR and 30 cycles for the secondary reaction denaturing at 94°C for 1 minute, annealing at 56°C for 2 minutes and extension at 72°C for 2 minutes, then a final extension step at 72°C for 5 minutes. The expected PCR product is 355bp. Gel electrophoresis was used to detect the amplified products on 2% agarose gel. Ten microlitres of PCR product was mixed with 5 µL of 5X loading buffer and electrophoresed at 120 Volts for 90 minutes. Then the agarose gel was stained with Ethidium Bromide to visualize and photograph the product on an UV. The PCR product size was estimated when compared with a 100bp DNA ladder.

S5 table. Number of parasite isolates with *pfkelch, pfcrt* mutations and gene amplification of *pfplasmepsin 2* before and after mass drug administration

**Countries**		***Pfkelch13***								***Pfplasmepsin2 CNVs***		***Pfcrt***	
		**E252Q**	**P441L**	**G538V**	**P574L**	**R575K**	**C580Y**	**A675V**	**WT**	**Single copy**	**Multiple copies**	**CVIET**	**CVIET+G353V**
Myanmar	Pre-MDA	0	0	0	1	0	0	0	0	121	0	7	0
	Post-MDA	2	2	2	2	1	0	2	13	36	0	9	0
Cambodia	Pre-MDA	0	0	0	0	0	19	0	0	10	11	4	7
	Post-MDA	0	0	0	0	0	3	0	0	1	1	1	7

Pfcrt was amplified from the DNA template using nested PCR covering exons 1 and 2 (amino acids 1 to 120) followed by sequencing. A PCR-restriction fragment length polymorphism assay was developed to assess previously identified *pfcrt* mutations [10] at positions N88K, T93S, H97Y, F145I, I218F, CVMNK72-76CVIET, N326S, M343I/L, G353V, I356T, and R371I. Only a single haplotype of CVIET at residues 72-76 and V at residue 353 was found in 7 isolates before and 7 after MDA in Cambodia, and none were found in Kayin, Myanmar. We were able to amplify pm2 only of two samples (of 7) because of insufficient DNA. We found that only one sample had multiple copies and the other had a single copy.
